# Alterations of SOCS1 and SOCS3 transcript levels, but not promoter methylation levels in subcutaneous adipose tissues in obese women

**DOI:** 10.1186/s12902-022-01247-5

**Published:** 2023-01-06

**Authors:** Solaleh Emamgholipour, Fataneh Esmaeili, Maryam Shabani, Seyedeh Zahra Hasanpour, Mahsa Pilehvari, Hossein Zabihi-Mahmoudabadi, Meysam Motevasseli, Mehrnoosh Shanaki

**Affiliations:** 1 Department of Clinical Biochemistry, School of Medicine, Tehran University of Medical Sciences, Tehran, Iran; 2 Student Scientific Research Center, Tehran University of Medical Sciences, Tehran, Iran; 3 Department of Anatomy, School of Medicine, Tehran University of Medical Science, Tehran, Iran Sciences, Tehran, Iran; 4 Department of Medical Laboratory Sciences, School of Allied Medical Sciences, Shahid Beheshti University of Medical Sciences, Tehran, Iran; 5 Department of Surgery, School of Medicine, Sina Hospital, Tehran University of Medical Sciences, Tehran, Iran; 6 Department of Medical Genetics, School of Medicine, Tehran University of Medical Sciences, Tehran, Iran

**Keywords:** SOCS1, SOCS3, Adipose tissue, Obesity, Insulin resistance, Expresseion, Methylation

## Abstract

**Background:**

Animal model studies suggest that change in the members of the suppressor of the cytokine signaling (SOCS) family (mainly SOCS1 and SOCS3) is linked to the pathogenesis of obesity-related metabolic disorders. Moreover, epigenetic modification is involved in the transcriptional regulation of the SOCS gene family. Here, we aimed to evaluate the mRNA expression as well as gene promoter methylation of SOCS1 and SOCS3 in subcutaneous adipose tissue (SAT) from obese women compared to normal-weight subjects. We also intend to identify the possible association of SOCS1 and SOCS3 transcript levels with metabolic parameters in the context of obesity.

**Methods:**

This study was conducted on women with obesity (*n* = 24) [body mass index (BMI) ≥ 30 kg/m ^2^] and women with normal-weight (*n* = 22) (BMI < 25 kg/m ^2^). Transcript levels of SOCS1 and SOCS3 were evaluated by real-time PCR in SAT from all participants. After bisulfite treatment of DNA, methylation-specific PCR was used to assess the putative methylation of 10 CpG sites in the promoter of SOCS1 and 13 CpG sites in SOCS3 in SAT from women with obesity and normal weight.

**Results:**

It was found that unlike SOCS3, which disclosed an elevating expression pattern, the expression level of SOCS1 was lower in the women with obesity as compared with their non‐obese counterparts (*P*-value = 0.03 for SOCS1 transcript level and *P*-value = 0.011 for SOCS3 transcript level). As for the analysis of promoter methylation, it was found that SOCS1 and SOCS3 methylation were not significantly different between the individuals with obesity and normal weight (*P*-value = 0.45 and *P*-value = 0.89). Correlation analysis indicated that the transcript level of SOCS1 mRNA expression had an inverse correlation with BMI, hs-CRP levels, HOMA-IR, and insulin levels. However, the SOCS3 transcript level showed a positive correlation with BMI, waist-to-height ratio, waist circumference, hip circumference, hs-CRP, HOMA-IR, insulin, fasting blood glucose, and total cholesterol. Interestingly, HOMA-IR is the predictor of the transcript level of SOCS1 (β =  − 0.448, *P*-value = 0.003) and SOCS3 (β = 0.465, *P*-value = 0.002) in SAT of all participants.

**Conclusions:**

Our findings point to alterations of SOCS1 and SOCS3 transcript levels, but not promoter methylation levels in subcutaneous adipose tissues from women with obesity. Moreover, mRNA expression of SOCS1 and SOCS3 in SAT was associated with known obesity indices, insulin resistance, and hs-CRP, suggesting the contribution of SOCS1 and SOCS3 in the pathogenesis of obesity-related metabolic abnormalities. However, further studies are required to establish this concept.

## Introduction

Human obesity, one of the serious health problems is becoming an epidemic all around the world [1, 2]. Obesity is a risk factor for some chronic disorders such as type 2 diabetes mellitus, cardiovascular diseases, and certain malignant conditions [1, 3]. Obesity is a complicated multifactorial condition that results from an imbalance between calorie intake and calorie consumption [4]. As an animate endocrine organ, adipose tissue has an important role in energy metabolism and regulating endocrine, metabolic, and immune responses [5]. Two main types of adipose tissue including subcutaneous adipose tissue (SAT) and visceral adipose tissue (VAT) have different cellular, molecular and clinical specifications. There is also evidence that SAT contributes more than VAT to the proinflammatory milieu linked to severe obesity [6] One of the most important outcomes of obesity is insulin resistance which is highly associated with the overproduction of pro-inflammatory cytokines produced by adipose tissue and results in chronic inflammation [7–9]. Over the years the members of the suppressor of cytokine signaling (SOCS) family (mainly SOCS1 and SOCS3) have received the lion’s share of the attention as key players in the development of insulin resistance.

SOCS family consists of eight members: SOCS 1–7 and cytokine-inducible SH2 protein (Cis) [10]. They are known as the regulator of cytokine signaling in different tissues in a cell-type-specific manner [11]. Each of which contains an amino-terminus end with limited homology, a central SH2 domain, and a conserved carboxyl-terminus domain (SOCS box). SOCS proteins can negatively modulate the signaling of cytokine receptors in several different ways. First, inhibiting Janus kinases directly. Second, compete for signaling molecules containing SH2-domains such as signal transducers and activators of transcription (STATs) for accessing receptor binding sites. Third, targeting receptor complex and related signaling proteins for proteasomal degradation by SOCS box [12]. Among SOCS family members SOCS1 and SOCS3 have received special attention in the pathogenesis of different disorders like immune disorders, tumorigenesis, type 2 diabetes, and obesity [13].

Increasing data from cell line studies and animal models point to the possible role of SOCS in the pathogenesis of obesity and related metabolic abnormalities. Lipopolysaccharide (LPS)-induced endotoxemia provides an experimental tool to assess the relationship between the effects of increased cytokine levels and insulin resistance. Ueki et al. found an increase in SOCS1, and SOCS3 proteins, in the liver, muscle, and, to a lesser extent, fat endotoxin-induced insulin resistance animal model. Moreover, they found that SOCS1 and SOCS3 inhibit insulin signaling and cause insulin resistance by inhibiting insulin receptor substrate 1 (IRS1) and IRS2 binding to the insulin receptor in cultured L6 myotubes and 3T3L1 adipocytes [14]. Also, there is evidence that SOCS3 deficiency in proopiomelanocortin neurons, as an important regulator of appetite and blood glucose, influences body weight regulation and energy balance in the setting of a control diet and a high-fat diet [15]. Furthermore, SOCS3 suppression prevented resistin, a well-known inflammatory adipocytokine, from antagonizing insulin action in 3T3-L1 adipocytes [16]. Moreover, overexpression of SOCS1 attenuates insulin-induced glycogen synthesis in myotubes and inhibits glucose uptake in 3T3L1 adipocytes [14].

Moreover, a trend of increase in circulating leptin levels was found in transgenic mice with muscle SOCS3 over-expression. This situation was similar to the scenario of insulin resistance due to impaired insulin signaling in insulin-sensitive tissue. Mechanistically, muscle SOCS3 overexpression in mice suppressed leptin-regulated genes involved in fatty acid oxidation and mitochondrial function and inhibited the effect of leptin on phosphorylation of alpha2 AMP-activated protein kinase (α2AMPK) and its downstream target, acetyl-CoA carboxylase (ACC). This data suggests that increased SOCS3 protein is sufficient to mediate insulin and leptin resistance in muscle in the context of obesity [17]. In some investigations, it is shown that increased BMI, body weight gain, and obesity are related to increased SOCS3 expression [17–19]. Also, it has been reported that SOCS1 expression is elevated in insulin-sensitive tissues in obese insulin-resistant db/db mice. Moreover, increased SOCS1 levels in cultured muscle cells and adipocytes can lead to insulin resistance via the inhibition of tyrosine phosphorylation of insulin receptor substrate proteins and subsequent downstream signaling [14]. In another study, adenoviral-mediated expression of SOCS1 in mouse liver blocked insulin signaling by ubiquitin-mediated degradation of insulin receptor substrates 1 and 2, resulting in insulin resistance [20]. Furthermore, SOCS1 gene promoter mutation is related to obesity and insulin resistance [21].

Recently, epigenetic alteration has been considered a process that may explain the etiology of complex disorders like obesity which is a result of the interaction between genetics and environmental conditions [22]. By definition, epigenetic regulation is heritable changes in a chromosome that affect the phenotype and gene expression with no effect on the DNA sequence [23]. One of the key types of epigenetic modification is DNA methylation which consists of adding one methyl group to cytosine at CpG islands and usually leads to suppression of gene expression [24]. Despite tremendous efforts to identify epigenetic contributions to obesity pathomechanism, there is still a great deal of uncertainty in this regard.

New emerging pieces of evidence suggest that aberrant DNA methylation is related to body weight and obesity [25–27]. The methylation status of SOCS1 and SOCS3 are evaluated in different cancers e.g., ovarian and breast carcinomas, Barrett’s adenocarcinoma, gastrointestinal cancers like hepatocellular carcinoma, pancreatic carcinoma, human lung, and head and neck cancer [28, 29]. To the best of the authors’ knowledge, no data were available on the alteration in both expression levels and methylation status of the gene promoter of SOCS1 and SOCS3 in human obesity. Here, we aimed to evaluate the mRNA expression as well as gene promoter methylation of SOCS1 and SOCS3 in SAT from obese women compared to normal-weight subjects. The study also intended to assess the possible association of SOCS1 and SOCS3 transcript levels with metabolic parameters in the context of obesity.

## Methods and patients

### Study participants

This case–control study protocol was approved by the ethics committee of Shahid Beheshti University of Medical Sciences (IR.SBMU.RETECH.REC.1396.961) in compliance with the principles of the Declaration of Helsinki. All methods were performed under the relevant guidelines and regulations.

Written informed consent was obtained from each individual before participation. The case group was selected among women with obesity (*n* = 22) [BMI ≥ 30 kg/m^2^] who were candidates for bariatric surgery (vertical sleeve gastrectomy and Roux-en-Y gastric bypass) at the bariatric surgery center of Erfan hospital. The control group was selected from normal-weight women(*n* = 24) [BMI < 25 kg/m^2^] who were under elective cholecystectomy or inguinal hernia at the center of advanced laparoscopic surgeries at Sina and Loqman Hakim hospitals, Tehran, Iran.

All participants were aged from 20 to 53 years and were selected from Iranian ethnic. The exclusion criteria were as follows: women with post-menopause, diabetes (Type 1 and/or 2), cardiovascular disease, liver disease, acute and chronic infectious diseases, autoimmune disease, malignancy, pregnancy, and taking drugs that can affect the metabolic parameters (e.g. metformin and statin). It should be noted that the participants were neither history of surgery or hospital admission during the last 6 months, nor smoking at the time of the study. However, one individual in women with obesity and two in the normal-weight group were taking levothyroxine and anti-hypertensive drugs at the time of the study, respectively. The anthropometric characteristics of all participants including weight, height, waist circumference (WC), and hip circumference were evaluated. Body mass index (BMI) was measured by dividing body weight in kilograms by height in meters squared (BMI = kg/m^2^). WC was assessed at the approximate midpoint between the lower margin of the last palpable rib and the top of the iliac crest using a flexible inch tape according to WHO guidelines. Hip circumference was measured at the level of the greatest protrusion of the buttocks while each individual stood erect with the feet together. Waist-to-hip ratio (WHR) and waist-to-height ratio(WHtR) were calculated by dividing WC in cm by hip and by height, respectively. The Systolic and diastolic blood pressure of participants was done three times after at least 15 min rest in a sitting position using a manual sphygmomanometer and the average of them was recorded.

### Blood collection and biochemical measurements

Venous blood samples of participants were collected after overnight fasting on the day of surgery as described previously [30, 31]. All blood samples were centrifuged at 2,000 g for 10 min at 4 °C. The serum was separated and transferred to the Eppendorf tube and immediately stored at -80 °C until the following analyses. Serum levels of fasting blood glucose (FBG), uric acid, urea, creatinine, high-density lipoprotein cholesterol (HDL-C), low-density lipoprotein cholesterol (LDL-C), triglyceride (TG), total cholesterol (TC), aspartate aminotransferase (AST), and alanine aminotransferase (ALT) were measured using commercial kits (Pars Azmoon, Tehran, Iran) on the auto analyzer (Roche, Cobas 6000 e501). Furthermore, high-sensitivity C-reactive protein (hs-CRP) was evaluated by an immunoturbidometric method using the Roche Integra analyzer. The fasting blood insulin was assessed using the ECL method by Cobas 6000 e601 auto analyzer. For the assessment of insulin resistance homeostasis model assessment of insulin resistance (HOMA-IR) was estimated using the following equation: fasting blood glucose (mg/dL) × fasting blood insulin (μU/mL) / 405 [30, 31].

### Adipose tissue samples

SAT specimens were obtained during the operation from the normal weight control group and obese participants. Briefly, subcutaneous fat (approximately 0.5 g) was obtained by cutting a small aperture under the skin using a scalpel blade. Adipose tissue biopsies were washed in sterile and cold phosphate-buffered saline, cut off into small pieces, and snap-frozen in liquid nitrogen. Then, the samples were kept at -80 °C until DNA and RNA extraction.

### CpG island prediction and promoter analysis

Using the RefSeq sequence of SOCS1 and SOCS3, all potential transcription start sites (TSSs) were identified. Then, 2,000 bp, including 1,000 bp extending from the 5’ upstream region to 1,000 bp downstream of each TSS, was used as input to the MethPrimer 2.0 online tool [32] for the prediction of CpG islands. The promoter position of genes was obtained from the eukaryotic promoter database (EPD) [33]. Looking for differences in methylation levels, 10 CpG sites in the promoter of SOCS1 and 13 in SOCS3 were analyzed. We used MSP primers from previous studies for this purpose [34].

### Distribution of CpG islands in selected regions of SOCS1 and SOCS3 genes

**Fig. 1:**
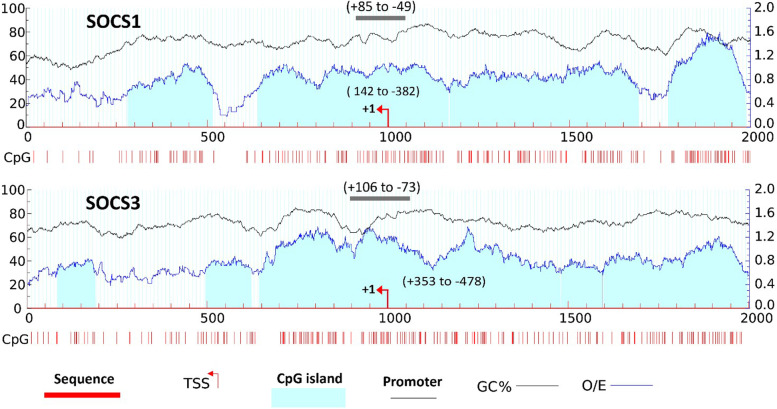
Bioinformatic analysis of SOCS1 and SOCS3 regulatory regions. The regulatory regions (grey) and CpG islands (light blue) are shown relative to the transcription start sites (+ 1). SOCS1, suppressor of cytokine signaling 1; SOCS3, suppressor of cytokine signaling 3

Using MethPrimer 2.0 online tool, 5 CpG islands around TSS-1 (NM_003955.5) and TSS-2 (NM_001378932.1), and 3 CpG islands around TSS-3 (NM_001378933.1) were predicted. In the case of SOCS1 (NM_003745.2), the number of predicted CpG islands was 4. Bioinformatic analysis of SOCS1 and SOCS3 regulatory regions was depicted in Fig. 1.

### Bisulfite treatment of DNA and methylation-specific polymerase chain reaction

Genomic DNA was extracted from SAT biopsy samples by The QIAamp Fast DNA Tissue Kit (QIAGEN). DNA bisulfite modification was performed based on the conversion of all the unmethylated cytosines to uracil while the methylated cytosines will be conserved using the EpiTect Fast Bisulfite Conversion kit (QIAGEN) following the manufacturer’s procedure. The modified DNA was used as a template for methylation-specific polymerase chain reaction (PCR) to demonstrate the promoter methylation of the selected CpG dinucleotides in the SOCS1 and SOCS3 regulatory regions. The assay was based on quantitative real-time PCR (RT-PCR) using specific primers set for methylated and unmethylated DNA sequences (M primers and U primers, respectively). Therefore, two PCRs were performed for each sample simultaneously using the M and U primer pairs. The methylated and unmethylated status of promoters was identified by successful amplification from the M primers and U primers. It should be noted that M and U primers had at least one CpG site at the 3’ end to maximal discrimination between methylated and unmethylated alleles. Primer sequences are illustrated in Table 1.

**Table 1:** Forward and reverse primers used for real-time PCR

Primer	Forward sequence	Reverse sequence
SOCS1	TTT TCG CCC TTAGCG TGA A	CAT CCA GGT GAA AGC GGC
SOCS3	GTC CCC CCA GAAGAG CCTATTA	TTG ACG GTC TTC CGA CAG AGA T
SOCS1M	GAGTATTCGCGTGTATTTTTAGG	CGACACAACTCCTACAACGACCG
SOCS1U	TGAGTATTTGTGTGTATTTTTAGG	CAACACAACTCCTACAACAACCA
SOCS3M	GGAGATTTTAGGTTTTCGGAATATTTC	CCCCCGAAACTACCTAAACGCCG
SOCS3U	GTTGGAGATTTTAGGTTTTTGGAATATTTT	AAACCCCCAAAACTACCTAAACACCA
Β-actin	TCCTTCCTGGGCATGGAGT	ACTGTGTTGGCGTACAGGTC

*M* Methylated, *U* unmethylated

The methylated level of DNA is expressed as the estimated amount of methylated DNA to the unmethylated DNA levels ratio calculated for each sample using the fluorescence threshold cycle. The efficiency for each single sample tube was estimated using the slope of the exponential phase as described previously [35]. Moreover, controls for unmethylated DNA and fully methylated DNA yielded 0% and 100% DNA methylation patterns used.

### RNA extraction and real-time PCR for quantitative assessment of mRNA expression

Frozen samples were homogenized in liquid nitrogen and total RNA was extracted from SAT by Hybrid-R™ kit (GeneAll). The purity and integrity of RNA were assessed by 260 nm to 280 nm ratio and gel electrophoresis, respectively. The complementary DNA (cDNA) synthesis was performed on 1000 ng of DNase-treated RNA using the Thermo Scientific RevertAid First Strand cDNA Synthesis kit. Quantitative Real-time PCR was accomplished by BioFACT™ 2X Real-Time PCR Master Mix (For SYBR Green I) in a Step-One-Plus TM real-time (ABI Applied Biosystems). β-actin was used as the reference gene. Primer sequences are illustrated in Table 1. Each sample was normalized to the corresponding value of the mean of the reference gene. For each sample, the difference in Ct values (ΔCt) between the target gene and the reference gene was calculated. The efficiency (E) of amplification for all target genes and reference genes ranged from 95 to 100% in all assays similar. To perform relative quantification 2^−ΔΔCt^ was applied.

### Statistical analysis

The normality of data was checked by the Shapiro–Wilk test and visually. Laboratory and anthropometric parameters with normal distribution are presented as mean ± standard deviation (SD), and data with skewed distribution were presented as median (interquartile ranges). The data comparison between cases and controls was performed with an independent samples t-test or Mann–Whitney U test, as appropriate. To remove the effects of potential confounders, an analysis of covariance (ANCOVA) was carried out. Log-transformation was employed for variables with non-normal distribution when being normally distributed outcomes were a necessary assumption to perform analysis. Correlation coefficients were calculated using the two-tailed Spearman’s correlation analysis. A stepwise multivariable linear regression analysis was performed to ascertain the best set of predictors for SOCS1 and SOCS3 gene expression. *P*-value < 0.05 was considered statistically significant. All data analysis was performed using SPSS 20 (SPSS, Chicago, IL, USA).

## Results

Clinical and laboratory characteristics of the women with normal weight and obesity are shown in Table 2. The mean age of cases and controls was 37.68 ± 9.07 and 34.92 ± 6.61 years, respectively (*P*-value = 0.241). Obesity indices including BMI, WC, hip, and WHtR were significantly higher in women with obesity in comparison to the controls. Furthermore, significantly higher circulating insulin levels, hs-CRP, and HbA1C, as well as HOMA-IR values, were observed in women with obesity Also, the obese group showed higher levels of creatinine, TC, and LDL-C compared to the normal-weight controls.

**Table 2:** The anthropometric, clinical, and metabolic characterizations of all participants

Variables	Normal-weight subjects (*n* = 22)	Obese subjects (*n* = 24)	*p*-value
Age (years)	37.68 ± 9.07	34.92 ± 6.61	0.241^a^
BMI (kg/m2)	23.31(22.81–24.34)	42.59(36.36–46.12)	0.000^b^
WC (cm)	85.00(82.25–87.25)	114.00(111.25–119.50)	0.000^b^
HC (cm)	95.00(90.00–97.00)	128.00(120.25–133.75)	0.000^b^
WHR,-	0.89(0.86–0.93)	0.92(0.87–0.94)	0.202^b^
WHtR,-	0.51 ± 0.04	0.72 ± 0.06	0.000^a^
SBP (mmHg)	120.00(110.00–120.00)	120.00(111.00–130.00)	0.266^b^
DBP (mmHg)	80.00(70.00–80.00)	80.00(70.00–90.00)	0.472^b^
FBG(mg/dL)	85.21 ± 7.29	89.00 ± 8.71	0.119 ^a^
Urea (mg/dL)	22.60 ± 7.54	26.53 ± 6.08	0.0573^a^
Creatinin(mg/dL)	0.58 ± 0.16	0.73 ± 0.11	0.001^a^
TG(mg/dL)	91.55(66.43–140.15)	105.95(66.55–152.35)	0.965^b^
TC(mg/dL)	147.40 ± 37.61	180.40 ± 25.48	0.001^a^
HDL-C(mg/dL)	44.02 ± 7.33	44.95 ± 72.87	0.666^a^
LDL-C(mg/dL)	88.70 ± 28.93	113.41 ± 19.65	0.001^a^
AST(mg/dL)	16.70(11.70–20.95)	20.65(16.20–23.95)	0.079^b^
ALT(mg/dL)	12.55(11.08–20.25)	20.30(15.35–30.20)	0.036^b^
hs.CRP(mg/dL)	1.80(0.98–2.56)	5.70(2.91–11.20)	0.000^b^
Insulin (µU/mL)	8.02 ± 3.57	19.4 ± 4.70	0.000^a^
HOMA-IR,-	1.69(1.03–2.06)	3.92(3.44–5.22)	0.000^b^
HbA1c,-	5.20(4.90–5.40)	5.50(5.13–5.68)	0.003^b^

Continuous variables with normal and non-normal distribution were described as the mean ± SD and median (IQR), respectively

*BMI* Body mass index, *WC* Waist circumference, *HC* Hip circumference, *WHR* Waist-to-hip ratio, *WHtR* Waist to Height Ratio, *SBP* Systolic blood pressure, *DBP* Diastolic blood pressure, *FBG* Fasting blood glucose, *TG* Triglycerides, *TC* Total cholesterol, *HDL-C* High-density lipoprotein cholesterol, *LDL-C* Low-density lipoprotein cholesterol, *AST* Aspartate aminotransferase, *ALT* Alanine aminotransferase, *hs-CRP* High-sensitivity C-reactive Protein, *AMH* Anti-mullerian hormone, *HOMA-IR* Homeostasis model assessment of insulin resistance, *HbA1c* Hemoglobin A1C

^a^ The *p*-value was determined with an independent samples t-test

^b^ The *p*-value was determined by Mann–Whitney U test

### SOCS1 and SOCS3 mRNA expression and promoter methylation in women with obesity and normal‑weight

**Fig. 2:**
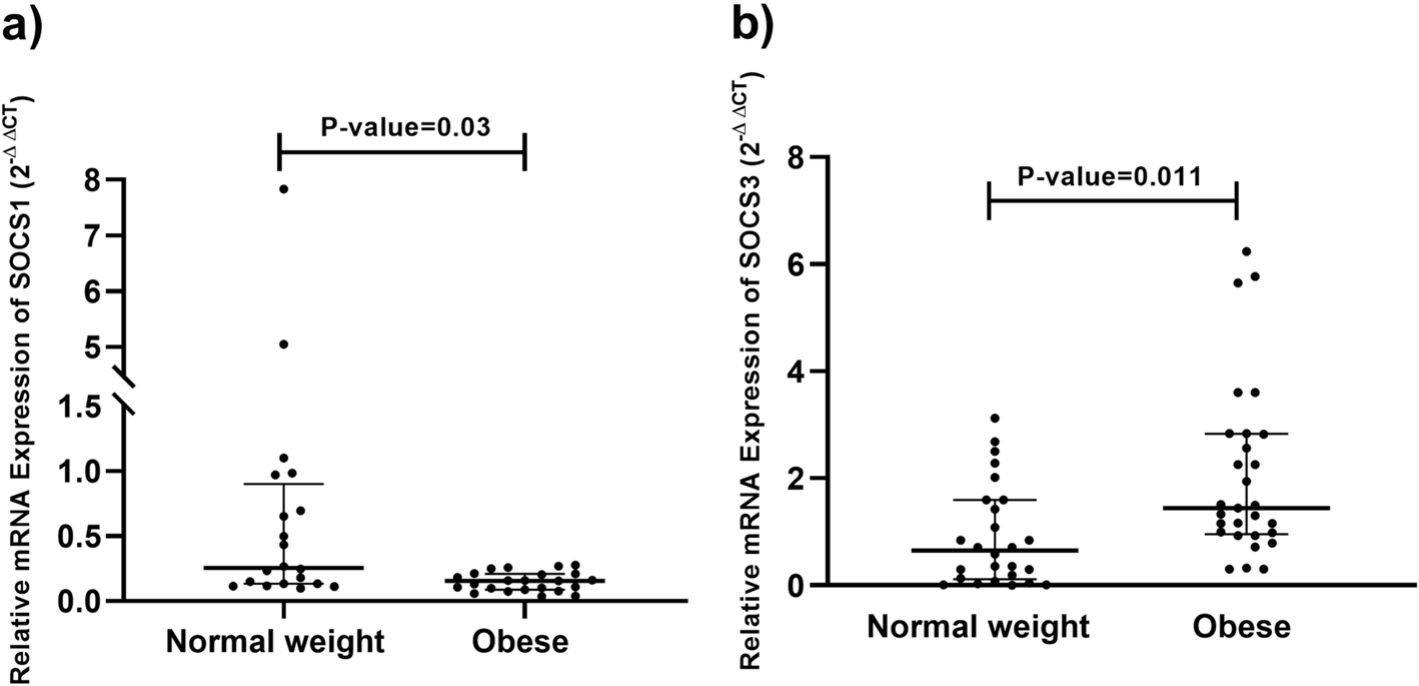
Expression of SOCS1 (**a**) and SOCS3 (**b**) genes in the subcutaneous adipose tissues (SAT) of women with obesity (O) and ones with normal weight (NW). Results, normalized to the corresponding value of housekeeping genes (β-actin), are shown as median (interquartile). Fold changes in gene expression of women with obesity relative to the controls were calculated by the 2-^ΔΔCt^ method. The p-value was determined by Mann–Whitney U test

The gene expression of SOCS1 and SOCS3 in adipose tissue of normal-weight and obese women is demonstrated in Fig. 2. In detail, SOCS1 gene expression was significantly lower in obese participants than in the normal-weight control group (*P*-value = 0.03, and Z-score = -2.167). While SOCS3 gene expression was markedly higher in the obese group than in control individuals (*P*-value = 0.011 and Z-score = -2.557). ANCOVA was performed to remove the age and drug effect on the SOCS1 and SOCS3 gene expression. The data revealed that SOCS1 and SOCS3 expression alteration was independent of age and drugs (*P*-value = 0.003 and *P*-value = 0.01, respectively).

**Fig. 3:**
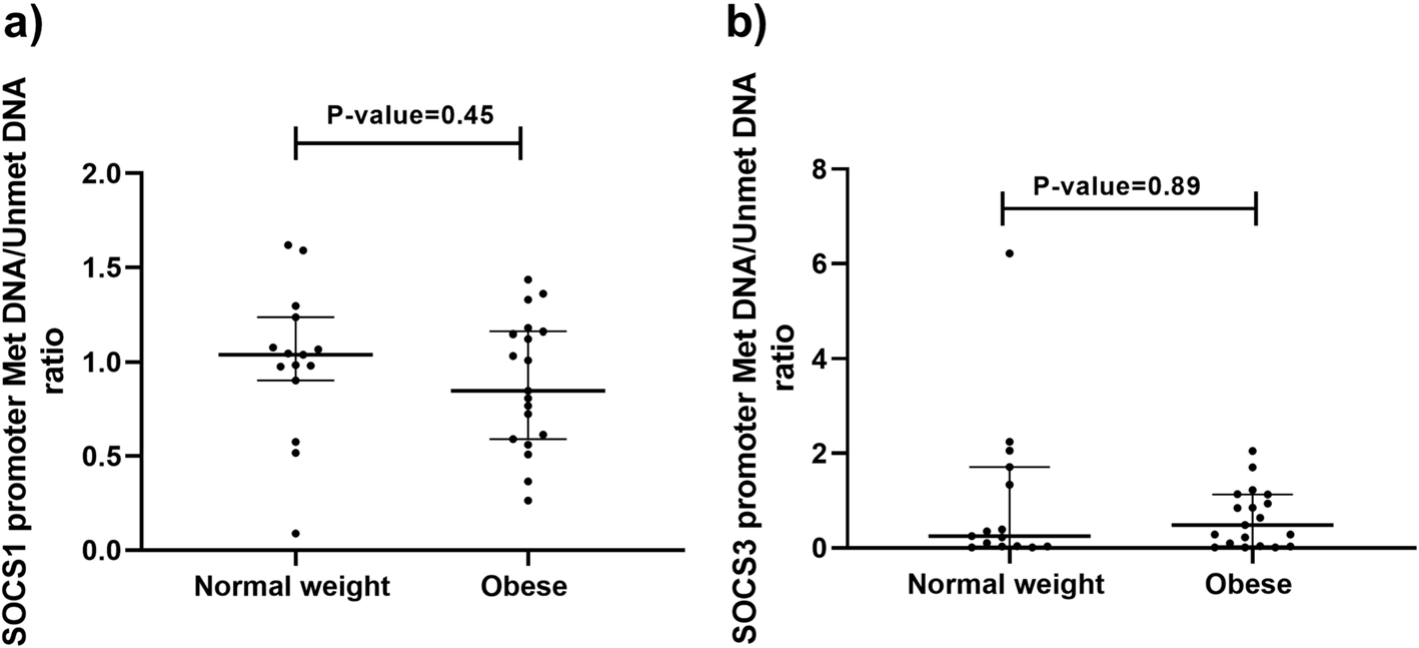
Methylation status of regulatory regions of SOCS1 (**a**) and SOCS3 (**b**) genes in the subcutaneous adipose tissues (SAT) of women with obesity (O) and ones with normal weight (NW). The methylated level of DNA is expressed as the estimated amount of methylated DNA to the unmethylated DNA levels ratio calculated for each sample using the fluorescence threshold cycle. The results were s expressed as fold change relative to the controls. Results were shown as median (interquartile ranges). The *p*-value was determined by Mann–Whitney U test

As for the analysis of promoter methylation, it was found that SOCS1 (*P*-value = 0.45 and Z-score = -0.78) and SOCS3 methylation (*P*-value = 0.89 and Z-score = -0.156) were not significantly different between the individuals with obesity and normal-weight (Fig. 3).

### Correlation of the SOCS1 and SOCS3 transcript levels with anthropometric and biochemical characteristics

Bivariate correlation analysis of SOCS1 and SOCS3 mRNA levels with anthropometric and laboratory characteristics in the whole population is demonstrated in Table 3. As demonstrated in Table 3, SOCS1 mRNA expression is inversely correlated with BMI (*r* = -0.334; *P*-value = 0.031), hs-CRP levels (*r* = -0.347; *P*-value = 0.024), HOMA-IR (*r* = -0.337; *P*-value = 0.029), and insulin levels (*r* = -0.367; *P*-value = 0.014). Moreover, the correlation between SOCS1 expression and waist-to-height ratio (*r* = -0.302; *P*-value = 0.052) as adiposity indices was marginally significant.

**Table 3:** The correlation of gene expression of SOCS1 and SOCS3 with anthropometric, and metabolic profiles in whole population study

	SOCS1 mRNA expression		SOCS3 mRNA expression	
	Spearman coefficient (r)	*p*-value	Spearman coefficient (r)	*p*-value
Age (years)	-0.007	0.963	-0.266	0.089
BMI (kg/m2)	-0.334	0.031	0.340	0.027
WC (cm)	-0.295	0.058	0.336	0.030
HC (cm)	-0.0276	0.077	0.374	0.015
WHR,-	0.053	0.740	0.022	0.891
WHtR,-	-0.302	0.052	0.392	0.010
SBP (mmHg)	-0.133	0.401	-0.100	0.530
DBP (mmHg)	0.279	0.073	0.025	0.887
FBG (mg/dL)	-0.073	0.646	0.392	0.010
TG (mg/dL)	0.077	0.627	0.120	0.450
TC (mg/dL)	-0.106	0.504	0.336	0.029
HDL-C (mg/dL)	-0.079	0.617	-0.166	0.292
LDL-C (mg/dL)	-0.199	0.206	0.304	0.050
hs.CRP (mg/dL)	-0.347	0.024	0.370	0.016
Insulin (µU/mL)	-0.367	0.014	0.361	0.019
HOMA-IR,-	-0.337	0.029	0.401	0.008

*BMI* Body mass index, *WC* Waist circumference, *HC* Hip circumference, *WHR* Waist-to-hip ratio, *WHtR* Waist to Height Ratio, *SBP* Systolic blood pressure, *DBP* Diastolic blood pressure, *FBG* Fasting blood glucose, *TG* Triglycerides, *TC* Total cholesterol, *HDL-C* High-density lipoprotein cholesterol, *LDL-C* Low-density lipoprotein cholesterol, *hs-CRP* High-sensitivity C-reactive Protein, *HOMA-IR* Homeostasis model assessment of insulin resistance

A stepwise linear regression analysis was performed to ascertain the best set of predictors for SOCS1 gene expression. Our results showed that HOMA-IR is the predictor of SOCS1 transcript level in SAT of all participants (β =  − 0.448, *P*-value = 0.003).

As for SOCS3 mRNA expression, we found that the transcript level of this member of the SOCS family had a significant positive correlation with BMI (*r* = 0.340; *P*-value = 0.027), WHtR (*r* = 392; *P*-value = 0.010), waist circumference (*r* = 0.336; *P*-value = 0.030), hip circumference (*r* = 0.374; *P*-value = 0.015), hs-CRP (*r* = 0.370; *P*-value = 0.016), HOMA-IR (*r* = 0.401; *P*-value = 0.008), insulin (*r* = 0.361; *P*-value = 0.019), FBG (*r* = 0.392, *P*-value = 0.010), and TC (*r* = 0.336; *P*-value = 0.029). Similar to SOCS1, HOMA-IR (β = 0.465, *P*-value = 0.002) was found to be the best predictor for SOCS3 mRNA expression in SAT of all participants following stepwise linear regression analysis.

## Discussion

Obesity is related to chronic inflammation and insulin resistance development. One of the plausible proteins involved is the SOCS family, especially SOCS1 and SOCS3 [9]. Data from in vitro studies and animal models point to the possible role of SOCS1 and SOCS3 in underlying mechanisms pertinent to obesity and associated metabolic disorders. The epigenetic regulation of transcriptional control by DNA methylation has gained an increasing interest in our understanding of obesity pathomechanism. Nowadays, enormous efforts are being made to investigate whether epigenetic factors occurring in the adipose tissue, like altered DNA methylation of promoter-associated CpG dinucleotides, are linked to metabolic abnormalities in the context of obesity-related disorders. Despite ample human studies on the alteration in SOCS1 and SOCS3 promoter methylation status and gene expression in various pathological conditions, no study has analyzed the alteration in SOCS1 and SOCS3 mRNA expression and their promoter DNA methylation in SAT of obese women compared to the non-obese control group.

In the present study, we observed that unlike SOCS1, which disclosed a decreased expression pattern, the transcript level of SOCS3 was higher in the obese group in comparison with the normal-weight one. Liver-specific SOCS3 knockout mice fed an HFD had elevated hypothalamic SOCS3, increased food intake, reduced energy expenditure, and increased lipogenic capacity of the liver which leads to steatosis, inflammation, and metabolic deterioration. There is evidence that systemic inflammation can lead to leptin resistance. Hence, elevated hypothalamic SOCS3 can contribute to leptin resistance in an HFD condition. SOCS3 inhibits insulin signaling and leads to the development of insulin resistance. Following binding to the insulin receptor, it prevents IRS association with insulin receptor substrate-1 (IRS1) and IRS2. Moreover, SOCS3 may also target IRS proteins for proteasomal degradation [9]. In line with our data, increased basal transcript levels of SOCS3 but not SOCS1 were found in peripheral blood mononuclear cells of individuals with obesity as compared with their non-obese counterparts. However, the mRNA level of SOCS3 and SOCS1 was lower in peripheral blood mononuclear cells from obese volunteers after stimulation with Toll-like receptor ligands [5]. In parallel, SOCS3 mRNA expression was significantly enhanced in type 2 diabetic patient skeletal muscle in comparison to the control subjects and is associated with reduced insulin-stimulated glucose uptake. Moreover, SOCS3 upregulation was found to be associated with insulin resistance and hyperglycemia in patients with diabetes [36].

Although the current study cannot address the underlying mechanism regarding the role of SOCS1 and SOCS3 in obesity and associated metabolic dysfunction, the possible mechanism can be derived from in vitro and murine model surveys.

Overexpression of SOCS3 in adipocytes from a transgenic mouse model (aP2- SOCS3 mouse) causes decreasing IRS1 protein levels and subsequent IRS1 and -2 phosphorylation, diminishing p85 binding to IRS1, and leads to decreased glucose uptake stimulated by insulin in adipocytes [7]. Moreover, SOCS3 deficiency enhances the phosphorylation of IRS1 and -2 stimulated by insulin and increases phosphatidylinositol 3 kinase (PI3K) activity, causes to increased insulin-stimulated glucose uptake in adipocytes [18]. There is also evidence that SOCS3-mediated insulin resistance is involved in the upregulation of mediators (e.g. tumor necrosis factor-α (TNF-α), Interleukin 6 (IL6)) signaling pathways. Additionally, the lack of SOCS3 limits the inhibitory effects of TNF-α on insulin signaling in adipocytes. Moreover, inhibiting SOCS3 production in adipose tissue of female mice can ameliorate whole-body insulin sensitivity in obesity [19].

In agreement with the above-mentioned data, SOCS3 mRNA expression in SAT showed a positive correlation with known obesity indices such as HOMA-IR, adiposity indices (e.g. BMI, WC, hip circumference), and hs-CRP (well-known inflammatory mediators). Hence, it can be speculated that increased expression of SOCS3 in adipose tissue from individuals with obesity can lead to metabolic abnormalities pertinent to obesity. However, the possible stimulatory role of insulin resistance, hyperinsulinemia, and inflammation in the induction of SOCS3 expression cannot be ignored. To support this notion, several hormones and cytokines are known to cause insulin resistance including insulin, growth hormone (GH), angiotensin II (AT-II), TNF-α, IL6, and interferon-gamma (IFN-γ) induce SOCS3 expression in cultured adipocytes [37, 38].

As for SOCS1, there has been a growing controversy in the available literature. For instance, macrophage deletion of SOCS1 augments sensitivity to lipopolysaccharide and palmitic acid resulting in systemic inflammation and hepatic insulin resistance [39]. *Emanuelli *et al*.* showed that SOCS1 knockout mice receiving a high-fat diet had more than 50% weight gain, increased fat mass as well as hepatic lipid content which was in parallel with increased inflammatory macrophage in white adipose tissue. This study showed that despite the role of SOCS proteins in obesity-related insulin resistance, SOCS1 deficiency alone may not be sufficient to alleviate high-fat diet-induced obesity and metabolic complications [40]. While SOC3 deficiency can protect against metabolic abnormalities and insulin resistance in HFD-induced obesity in mice [9]. Above mentioned data can partly support findings regarding the lower expression of SOCS1 in obese women in comparison with women with normal weight as well as an inverse correlation of SOCS1 mRNA expression BMI, hs-CRP levels, HOMA-IR, and insulin levels. However, some studies showed inconsistent results. For instance, *Kawazoe *et al*.* reported that SOCS1 overexpression reduces insulin-stimulated IRS1 phosphorylation [41]. Also, SOCS1 has been shown to down-regulate insulin signaling and cause insulin resistance [42]. One study that was conducted on 30 obese and 30 non-obese subjects demonstrated that basal expression of SOCS1 expressions in peripheral blood mononuclear cells (PBMCs) was similar in obese and non-obese groups [5].

Next, we investigated DNA methylation levels of the SOCS1 and SOCS3 promoter in SAT to explore if altered transcript levels of SOCS1 and SOCS3 promoter DNA methylation can be primarily ascribed to a transcriptional regulation alteration.

Our results showed that neither SOCS1 methylation level nor SOCS3 methylation level alter in SAT women with obesity in comparison with ones with normal weight. Although methylation levels in regulatory regions of genes may differ between tissues, Andia et al. reported DNA methylation levels for SOCS1 and SOCS3 did not differ between microdissected gingival tissue from patients with and without a history of periodontitis [43].

Conversely, other studies showed that SOCS1 promoter methylation is associated with several conditions such as liver [44] and gastric [45] cancer, multiple myeloma [46], myeloproliferative neoplasm [47], lymphoma [48], pancreatic ductal neoplasms [49] and development of in-stent restenosis (ISR) after percutaneous coronary intervention [50]. As for SOCS3 methylation, aberrant methylation in the promoter region of this gene has been observed in several human malignancies [34, 51, 52].

Although more detailed surveys are needed to explain our findings regarding no change in the methylation level of SOCS1 and SOCS3, several possible reasons should be considered in this regard as stated below:

WAT is composed of a heterogeneous cellular population including mature adipocytes and other cells of the stromal vascular fraction such as preadipocytes, blood cells, endothelial cells, and a range of inflammatory leukocytes [53]. There is also evidence that epigenetic mechanisms contribute to the maintenance of site-specific gene expression patterns in WAT. For instance, pre-adipocytes seem to retain an intrinsic epigenetic memory of their regional location in the body [54]. In the present study, we investigated the promoter methylation of SOCS1 and SOCS3 in WAT, however, the various cell type located in WAT has a discrete epigenetic profile and subsequently different expression profile. Hence, an assessment of promoter methylation in isolated cells from WAT is suggested to gain more insights into the possible role of promoter methylation in the context of obesity.

Moreover, a wide range of epigenetic modifications is involved in the transcriptional regulation of the SOCS gene family [55–57]. Here, we only select promoter methylation as one of the possible mechanisms, therefore, investigation of other epigenetic regulatory mechanisms is warranted in this regard.

Although the current study as a preliminary one opens an avenue to the involvement of SOCS1 and SOCS3 in the obesity etiology, however, several limitations merit comment. Firstly, the current study has a cross-sectional design which limits us to determine the causal relationship between the variables. Secondly, assessment of gene expression and promoter methylation in visceral adipose tissue can provide valuable information on the role of the SOCS gene family in the setting of obesity. Thirdly, more studies with a larger sample size are necessary to unravel the exact role of SOCS 1 and SOCS3 in obesity-associated metabolic abnormalities.

In summary, we provide evidence regarding alterations of SOCS1 and SOCS3 transcript levels, but not promoter methylation levels in subcutaneous adipose tissues in obese women. Moreover, mRNA expression of SOCS1 and SOCS3 in SAT was associated with known obesity indices, insulin resistance, and hs-CRP, suggesting the contribution of SOCS1 and SOCS3 in the pathogenesis of obesity-related metabolic abnormalities. However, further studies are required to establish this concept.

## Data Availability

The datasets used and/or analyzed during the current study are available from the corresponding author upon reasonable request.

## Abbreviations

SAT: Subcutaneous adipose tissue
VAT: Visceral adipose tissue
SOCS: Suppressor of cytokine signaling
Cis: Cytokine-inducible SH2 protein
STATs: Signal transducers and activators of transcription
IRS1: Insulin receptor substrate 1
BMI: Body mass index
WC: Waist circumference
HC: Hip circumference
FBG: Fasting blood glucose
HDL-C: High-density lipoprotein cholesterol
LDL-C: Low-density lipoprotein cholesterol
TG: Triglyceride
TC: Total cholesterol
VLDL: Very-low density lipoprotein cholesterol
AST: Aspartate aminotransferase
ALT: Alanine aminotransferase
hs-CRP: High sensitivity C-reactive protein
HOMA-IR: Homeostasis model assessment of insulin resistance
PCR: Polymerase chain reaction
RT-PCR: Real-time polymerase chain reaction
cDNA: Complementary DNA
WHtR: Waist-to-height ratio
WHR: Waist-to-hip ratio
SBP: Systolic blood pressure
DBP: Diastolic blood pressure
HA1C: Hemoglobin A1C
GH: Growth hormone
AT-II: Angiotensin II
TNF-α: Tumor necrosis factor-α
IL6: Interleukin-6
IFN-γ: Interferon-gamma
HFD: High-fat diet
